# The unconditioned fear response in dystrophin-deficient mice is associated with adrenal and vascular function

**DOI:** 10.1038/s41598-023-32163-w

**Published:** 2023-04-04

**Authors:** Angus Lindsay, Aaron P. Russell

**Affiliations:** Institute for Physical Activity and Nutrition, School of Exercise and Nutrition Sciences, Deakin University, Geelong, Australia

**Keywords:** Neuromuscular disease, Behavioural genetics

## Abstract

Loss of function mutations in the gene encoding dystrophin elicits a hypersensitive fear response in mice and humans. In the dystrophin-deficient *mdx* mouse, this behaviour is partially protected by oestrogen, but the mechanistic basis for this protection is unknown. Here, we show that female *mdx* mice remain normotensive during restraint stress compared to a hypotensive and hypertensive response in male *mdx* and male/female wildtype mice, respectively. Partial dystrophin expression in female *mdx* mice (heterozygous) also elicited a hypertensive response. Ovariectomized (OVX) female *mdx* mice were used to explain the normotensive response to stress. OVX lowered skeletal muscle mass and lowered the adrenal mass and zona glomerulosa area (aldosterone synthesis) in female *mdx* mice. During a restraint stress, OVX dampened aldosterone synthesis and lowered the corticosterone:11-dehydrocorticosterone. All OVX-induced changes were restored with replacement of oestradiol, except that oestradiol lowered the zona fasciculata area of the adrenal gland, dampened corticosterone synthesis but increased cortisol synthesis. These data suggest that oestrogen partially attenuates the unconditioned fear response in *mdx* mice via adrenal and vascular function. It also suggests that partial dystrophin restoration in a dystrophin-deficient vertebrate is an effective approach to develop an appropriate hypertensive response to stress.

## Introduction

Brain dystrophin isoforms (Dp427, Dp260, Dp140, Dp116 and Dp71/Dp40) are transcribed in a tissue-dependent manner and perform several crucial processes^1,2^, such as regulating cerebral volume^3^, cellular stability via the dystrophin-glycoprotein-complex (DGC)^4^ and synaptic transmission^5^. A mutation in the gene encoding dystrophin (DMD gene) inhibits the expression and compromises the function of dystrophin. This disrupts various functions in the central nervous system (CNS). The severity of CNS disruption is associated with the location of the DMD gene mutation. For example, mutations in the Dp427 and Dp140/Dp71 variants are implicated in the severity of neurocognitive disabilities in humans affected by the terminal neuromuscular disease, Duchenne muscular dystrophy (DMD)^6^.

DMD is a X-linked disease affecting 1 in 5000 males^7^. DMD causes severe striated muscle degeneration that compromises the strength and capacity of the contractile tissue resulting in premature death due to cardiorespiratory failure. Up to 44% of patients with DMD suffer from brain-related co-morbidities^8^. These co-morbidities impact social development^9,10^ and associate with neurocognitive disability and worse prognostic outcomes^11^. Neurocognitive disabilities range from lower IQ, verbal and memory abnormalities, attention deficit hyperactivity disorder, anxiety, depression and autism^12^. In patients with DMD stress-inducing auditory and visual stimuli cause heightened levels of physiological anxiety, including fluctuations in heart rate and skin conductance compared to age-matched peers^13^. The intensity of physiological anxiety to stressful stimuli is correlated with the absence or expression of Dp140^13^.

The *mdx* mouse model of DMD does not express the Dp427 isoform due to a point mutation in exon 23^14^, causing it to suffer from muscle degeneration and a plethora of neurocognitive disabilities^15,16^. When challenged by a spectrum of stressful stimuli, a dysfunctional fear or threat response causes acute and sustained physical inactivity^5,17,18^, exacerbation of some dystrophinopathy phenotypes^19,20^ and cardiac failure due to hypotension^21^. This dysfunctional fear or threat response in *mdx* mice is driven by brain^5,22^ and skeletal muscle-sepcific dystrophin^21,23^. This is also associated with gamma-aminobutyric acid (GABA) receptor expression and activity^5^ and blood pressure^21^, respectively. During a stressor, *mdx* mice hyperactivate their hypothalamic–pituitary–adrenal (HPA)^20,21^ and renin–angiotensin–aldosterone (RAAS) axes^20^; a response significantly greater in female relative to male *mdx* mice^20^. Super-activation of the HPA and RAAS axis in female *mdx* mice is associated with greater resistance to stressful stimuli. Interestingly, this behaviour is abolished after ovariectomy and partially rescued with the replacement of oestradiol (E2)^20^.

The mechanisms controlling female sex hormone-dependent regulation of the stress response in *mdx* mice is unknown. Therefore, in study 1, we aimed to determine if blood pressure control can explain the difference in the dysfunctional fear or threat response of male and female *mdx* mice and if 30–60% dystrophin expression can rescue the dysfunctional fear or threat response in female *mdx* mice (heterozygous mice). In study 2, we aimed to understand how oestrogen regulates the dysfunctional fear or threat response in female *mdx* mice.

## Materials and methods

### Study 1

#### Experimental mice

Seven-week-old male and female C57BL/10ScSn-*Dmd*^*mdx*^/Arc (male *mdx*-hemi and female *mdx*-homo, respectively) and C57BL/10ScSn mice (wildtype; WT) were purchased from Animal Resources Centre (ARC; Perth, WA, Australia). Female heterozygous *mdx* mice (female *mdx*-het) were generated by mating male WT with *mdx*-homo in-house and genotyping was performed by Transnetyx (Cordova, TN, USA) on all female offspring. All mice were housed in groups of four per cage on a 12/12 h light/dark cycle with food and water provided ad libitum. *Mdx* mice were used as an experimental model of human DMD because they have a similar dysfunctional fear or threat response to *mdx*52 mice, which lack Dp427 and Dp140^24^. We previously showed that the fear or threat response in *mdx* mice is highly variable and associated with disease pathogenesis^20^. However, to ensure the biological variability of the behavioural responses were captured, we used large sample sizes and set no inclusion or exclusion criteria for the study.

#### Experimental design

Mice acclimated for 1 week prior to completing any experiment. At 8-weeks of age, male WT (n = 49), female WT (n = 42), female *mdx*-het (n = 77), male *mdx*-hemi (n = 119) and female *mdx*-homo (n = 117) mice completed three behavioural stressors, each separated by 1 week: forced downhill treadmill exercise, scruff-restraint and plexiglass-restraint (tube restraint; blood pressure analysis). Forced downhill treadmill exercise and scruff-restraint were accompanied by monitoring of physical activity before and immediately after the intervention. Male *mdx*-hemi and female *mdx*-homo mice also completed each stressor on two more occasions, each 1 week apart to assess habituation. For each genotype, a cage was selected randomly from the pool of all cages for completion of the stressors. Although time of day does not impact the behavioural response of *mdx* mice to stress^20^, testing order of mice was randomized daily. At the conclusion of the final plexiglass-restraint, all mice were killed by CO_2_ asphyxiation and cervical dislocation. Researchers were aware of mouse genotype throughout the conduction of experiments and data analyses.

#### Treadmill exercise

Mice were placed into an activity monitoring chamber to assess physical activity as previously described^20^. Mice were then run four at a time and the lanes of the treadmill were cleaned between each test. The treadmill was set at 0 m/min for two min before increasing to 10 m/min for 1 min. The treadmill increased by 1 m/min until 15 m/min was reached, which was then maintained for 15 min. At the completion of exercise, mice were again placed into a clean and sterilized activity monitoring chamber to assess physical activity.

#### Scruff-restraint

Mice were placed into an activity monitoring chamber to assess physical activity. Mice were then grasped by the nape between the thumb and index finger. The tail was placed between the fourth and fifth digit and the mouse was inverted such that it was in a supine orientation. The mouse remained in this position for 30 s. At the completion of the scruff-restraint, mice were again placed into a clean and sterilized activity monitoring chamber to assess physical activity because scent impacts activity of *mdx* mice following a stressor^17^.

#### Plexiglass-restraint (blood pressure)

Mice were placed into the CODA non-invasive blood pressure system for 5 min using the 25–50 g mouse holder (Kent Scientific, Torrington, CT, USA). The plexiglass restraint prevented the mouse moving in any direction and was adjusted to accommodate the size of each mouse. The restraint was placed on a 37 °C warming platform and a mouse sized tail cuff (8–75 g mouse) was placed on the tail, ensuring only the tip of the tail was visible for consistency across the cohorts. Blood pressure was automatically assessed every 30 s for 5 min (10 total measurement) using volume pressure recording (VPR) sensor technology and in-built CODA software. Mean arterial pressure as well as the shock index (maximum heart rate/low systolic blood pressure; index of hypovolemic stress) was used to assess stress sensitivity in *mdx* mice.

#### Physical activity monitoring

Physical activity was monitored using an Accuscan system by Omnitech Electronics, Inc. (Columbus, OH, USA). Physical activity was measured in the x, y and z planes using horizontal and vertical beam breaks. Mice were monitored four at one time and activity monitors were cleaned and sterilized prior to a mouse being placed in the chamber. Movement time (MT; s), vertical activity (VA; mouse rearing on its hind legs; counts), distance ambulated (DA; m), average velocity (m/s) and peak velocity (m/s) were used to assess physical activity levels. Data are either presented as the ratio of post-stressor physical activity compared to pre-stressor physical activity (% of intervention) or as a freezing % (total time spent immobile during the 5 min post-stress). Greater physical activity levels is associated with increased levels of resistance to behavioural stressors^21^.

### Study 2

#### Experimental mice and design

Eight-week-old C57BL/10ScSn-*Dmd*^*mdx*^/Arc female mice (female *mdx*-homo) following a sham, ovariectomy (OVX) or OVX + E2 supplementation were purchased from the ARC (n = 15/group). The physical activity data in response to a series of behavioural stressors was previously published in these experimental mice^20^. All mice were housed in groups of four per cage on a 12/12 h light/dark cycle with food and water provided ad libitum. Mice acclimated for 1 week prior to completing a scruff restraint and forced downhill treadmill exercise test, followed 1 week later by another scruff restraint. Fifteen minutes later, serum and tissue were collected after CO_2_ asphyxiation and cervical dislocation. The order of testing was randomized both within and between each experimental group and researchers were aware of group allocation throughout the conduction of experiments and data analyses.

#### OVX and E2 pellet implantation

Mice (8-weeks-old) were anesthetized with an intraperitoneal injection of 75–100 mg/kg ketamine/xylazine (0.1 mL per 10 g mouse body mass). While anesthetized, mice were placed on a warming mat in ventral recumbency, injected subcutaneously with 0.05 mg/kg buprenorphine, hair removed from both flanks from the caudal rib to the femur, the surgical site disinfected with warmed chlorhexidine and a dorsoventral incision was made in the paralumbar fossa mid-way between the caudal rib and femur on both sides of the mouse through the skin and muscle wall. The ovary and oviduct were exteriorized through the incision and either removed by cauterizing between the uterine horn and the oviduct and cutting through the periovarian fat pad (OVX) or returned into the peritoneal cavity (sham). The muscle wall was then closed with absorbable suture and a 0.5 mL lignocaine (5 mg/kg) and bupivacaine (1.2 mg/kg) splash block administered to the incision site. A placebo (60-day release; 0.18 mg) or E2 pellet (60-day release; 0.18 mg; 3.4 µg/day) were then placed under the skin at the surgical site (Innovative Research of America, Sarasota, FL, USA). E2 concentrations reflect physiological hormone levels in mice^25^. Sterile wound clips closed the skin incision, mice received a 5 mg/kg subcutaneous carprofen injection, and 0.5 mL warmed lactated ringers solution subcutaneously. Supplemental heat was provided during recovery and animals were placed in home cages with access to floor feed, moisture sources and nesting materials. Wound clips were removed after 7–10 days and mice were shipped 2 weeks following surgery.

#### Physical activity monitoring

Performed according to Study 1.

#### Tissue collection

Tissue collection was completed according to our previous methods^20^. Mice were killed by CO_2_ asphyxiation followed by cervical dislocation. Trunk blood was collected by decapitating the mouse immediately after cervical dislocation and collecting into a sterile tube. Blood was allowed to clot for 30 min at room temperature before centrifugation at 20,000*g* for 10 min. Serum was removed and snap frozen in liquid nitrogen and stored at − 80 °C until analyses. Tibialis anterior (TA), diaphragm and adrenal glands were collected and frozen in liquid nitrogen cooled isopentane before storing at − 80 °C until histological analyses.

#### Histology

Tissue sections (8 µm) were stained with H&E for adrenal gland and skeletal muscle pathology (n = 5–8/stress group) as previously described^26^.

#### Collagen content

Tibialis anterior and diaphragm sections (8 µm mid-belly cryosections) were stained using the Trichrome Stain Kit according to the manufacturer’s instructions (Ab150686, Abcam, Sydney, Australia).

#### Visualization

All muscle and adrenal gland sections were visualized using a Nikon Eclipse Ti2 microscope (Nikon, Australia) and captured using a Nikon DS-Qi2 camera, powered by Nikon Elements software (Nikon). Adrenal and TA cross-sectional area in millimeters squared were measured by tracing the section border in ImageJ (NIH, Bethesda, MD). Central nucleated fibres in the TA (400–600 fibers; 3 sections located in the upper, middle and lower quadrants of the cross-section) and diaphragm (200–300 fibers; 3 sections located in the upper, middle and lower quadrants of the cross-section) were quantified in ImageJ. Diaphragm thickness was measured in ImageJ by taking an average of five measurements equally spaced along the length of the muscle section. Collagen content was determined using the colour thresholding function in ImageJ.

#### Metabolomics

Samples were extracted and analysed by LC–MS as previously described^20^*.* Sample extraction: serum samples were thawed on ice and 10 µL was aliquoted for analysis by liquid chromatography-mass spectrometry (LC–MS) by Metabolomics Australia (Bio21 Molecular Science and Biotechnology Institute, University of Melbourne, Parkville, Australia). Samples were extracted using 180 µL 1:1 acetonitrile/methanol solution containing 2 µM 13C-sorbitol, 2 µM 13C, 15N-AMP and 2 µM 13C15N-UMP as internal standards. An additional 10 µL from each of the 40 serum samples was pooled for serum quality control (SQC) samples (10 µL), which were extracted with the serum samples. Samples were prepared in batches of 24, with a PQC extracted every ~ 10 sample. Samples were then vortexed for 30 s, incubated for 10 min at 4 °C using an Eppendorf Thermomixer and centrifuged at max speed for 10 min at 4 °C. After centrifugation, 60 µL of supernatant was transferred into a glass HPLC insert and 10 µL of the supernatant was pooled to create a pooled biological quality control (pbQC), which were all pooled post extraction and run as pbQC in the batches. LC–MS: Samples were stored in an autosampler at 4 °C, polar metabolites were separated by injecting a 10 μL sample onto a SeQuant ZIC-pHILIC column (150 mm × 4.6 mm, 5 μm) maintained at 30 °C using Solvent A (20 mM (NH_4_)_2_CO_3_, pH 9.0 (Sigma-Aldrich) and Solvent B (100% acetonitrile) at a flow rate of 300 μL/min and analysed on an Agilent 1200 series HPLC system (Agilent Technologies). The gradients used were: time (t) = 0 min, 80% B; t = 0.5 min, 80% B; t = 15.5 min, 50% B; t = 17.5 min, 30% B; t = 18.5 min, 5% B, t = 21.0 min, 5% B; t = 23 min, 80% B. The mass spectrometry analysis was performed on an Agilent 6545 series quadrupole time-of-flight mass spectrometer (QTOF MS) (Agilent Technologies). The LC flow was directed to an electrospray ionization source (ESI), where metabolite ionization in negative mode was performed with a capillary voltage of 2500 V, a drying gas (N_2_) pressure of 20 psi with a gas flow rate of 10.0 L/min, a gas temperature in the capillary of 325 °C and fragmentor and skimmer cap voltages of 125 V and 45 V, respectively. LC/MS data was collected in centroid mode with a scan range of 60–1200 m/z and an acquisition rate of 1.5 spectra/s in all-ion fragmentor (AIF) mode, which included three collision energies (0, 10, 20 V). Prior to analysis, mass calibration was performed for negative mode to 0.5 ppm accuracy of the m/z value. Internal mass calibration was performed using Agilent ESI-TOF Reference Mass Solution containing purine (119.036320) and hexakis (1H,1H,3H-tetrafluoropropoxy) phosphazine (981.99509) (API-TOF Reference Mass Solution Kit, Agilent Technologies), which was continuously flowed into the ESI source at a flow rate of 200 μL/min.

#### Corticosterone ELISA

Serum was analysed according to the manufacturer’s instructions (K014-H1, Arbor Assays, Ann Arbor, MI, USA).

#### Statistics

All analyses were conducted in GraphPad Prism 8.0.0 and there were no animal exclusions. Physical activity, blood pressure, metabolomics and muscle pathology were compared using either a one-way or two-way ANOVA and *post-hoc* Tukey’s test after checking for data normality. When data were not normally distributed, a Kruskal–Wallis test or Friedmann’s test were performed with Dunn’s *post-hoc* analysis, respectively. Linear regression was performed for correlative analyses between freezing time after treadmill exercise, scruff-restraint and mean arterial pressure. Data are presented as mean ± SD with significance set as p < 0.05.

### Ethical approval

All studies were reviewed and approved by the Deakin University Animal Ethics Committee (G04-2020 and G09-2020). All animals were housed and treated in accordance with standards set by Deakin University Animal Welfare Committee, which complies with the ethical and governing principles outlined in the Australian code for the care and use of animals for scientific purposes. The study is reported in accordance with ARRIVE guidelines.

## Results

### Study 1

**Figure 1:**
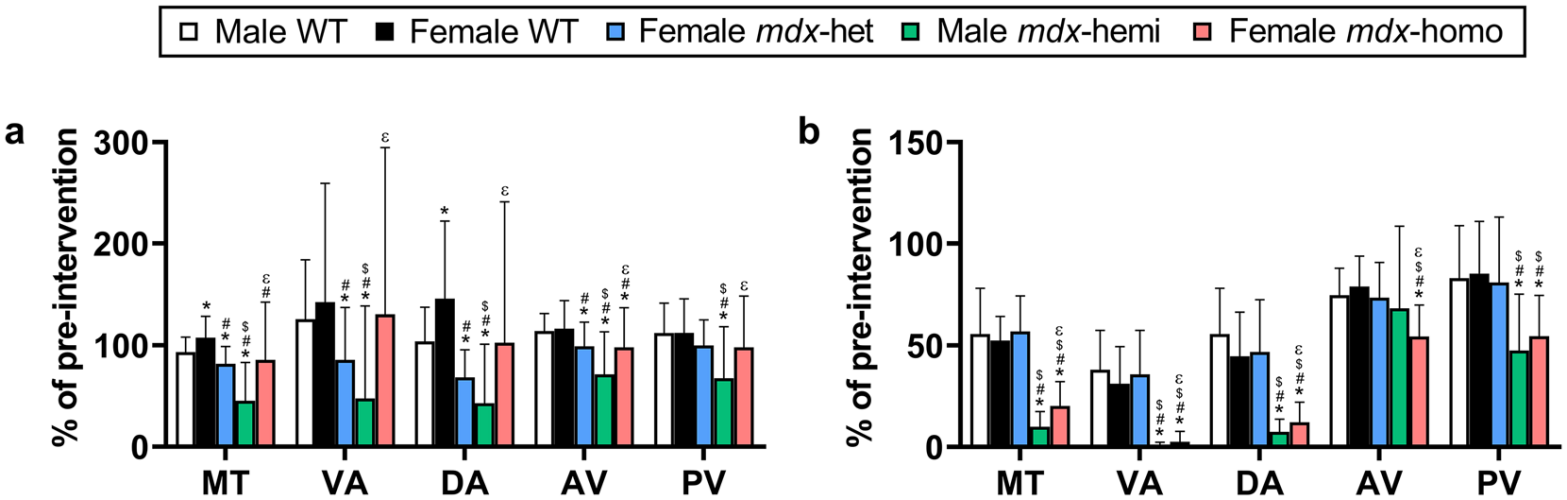
Partial dystrophin expression desensitizes *mdx* mice to behavioural stress. (**a**) Physical activity in response to forced downhill treadmill exercise and (**b**) a 30 s scruff-restraint. Data are presented as the ratio of post-stressor physical activity to pre-stressor physical activity (% of intervention). *WT* wildtype, *het* heterozygous, *hemi* hemizygous, *homo* homozygous, *MT* movement time (s), *VA* vertical activity (counts), *DA* distance ambulated (m), *AV* average velocity (m/s), *PV* peak velocity (m/s). Male WT (n = 49), female WT (n = 42), *mdx*-het (n = 77), *mdx*-hemi (n = 119) and *mdx*-homo (n = 117). Data are mean ± SD. *Different from male WT, ^#^different from female WT, ^$^different from female *mdx*-het, ^ε^different from male *mdx*-hemi. Data were analysed using a two-way ANOVA with a Tukey’s *post-hoc* analysis. Significance was set at p < 0.05.

There was a genotype effect for the level of physical activity following forced downhill treadmill exercise (Fig. 1a; p < 0.001) and scruff-restraint (Fig. 1b; p < 0.001). In response to treadmill exercise, female WT mice were more active than male WT mice (p = 0.030) and female *mdx*-het mice were less active relative to WT mice of both sexes across multiple indices of physical activity (p < 0.001). Male *mdx*-hemi mice were less active compared to all other genotypes (p < 0.001) while female *mdx*-homo mice were less active relative to female WT mice (p = 0.009) and more active than female *mdx*-het mice (p = 0.020). In response to scruff-restraint, there was no difference in physical activity between male WT, female WT and female *mdx*-het mice (p ≥ 0.773). Male *mdx*-hemi and female *mdx*-homo mice were less active across all indices of physical activity relative to male WT, female WT and female *mdx*-het mice (p < 0.001) but male *mdx*-hemi mice were less active than female *mdx*-homo mice across four of the five physical activity parameters (p = 0.013 to < 0.001).

**Figure 2:**
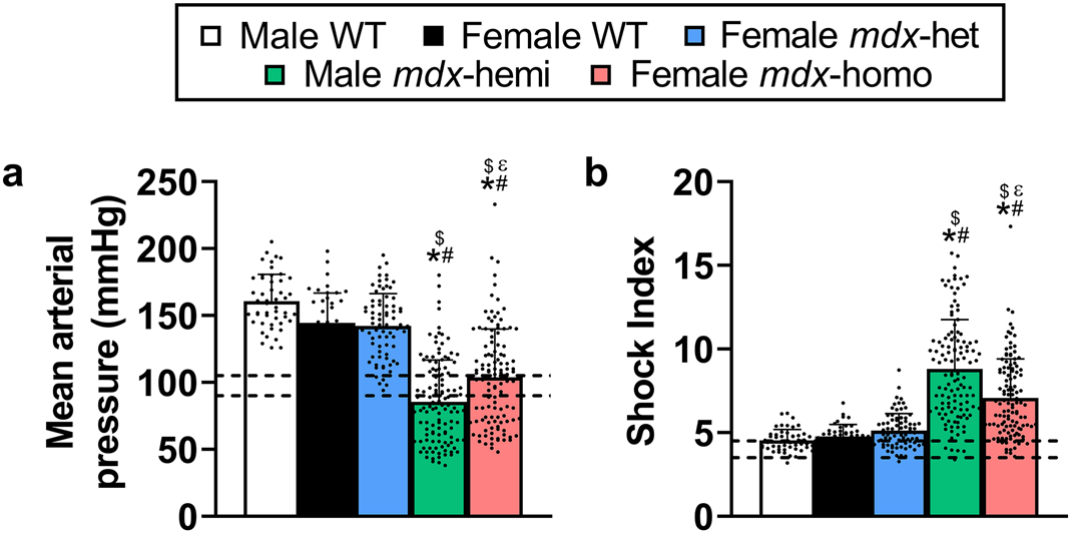
Partial dystrophin expression elicits a hypertensive response to stress in *mdx* mice. (**a**) Mean arterial pressure and (**b**) shock index during a 5 min plexiglass-restraint stress. WT; wildtype, het; heterozygous, hemi; hemizygous, homo; homozygous and shock index; heart rate/systolic pressure. Male WT (n = 49), female WT (n = 42), *mdx*-het (n = 77), *mdx*-hemi (n = 119) and *mdx*-homo (n = 117). Data are mean ± SD. --- Basal mean arterial pressure and shock index range for WT and *mdx* mice according to Razzoli et al.^21^. *Different from male WT, ^#^different from female WT, ^$^different from female *mdx*-het, ^ε^different from male *mdx*-hemi. Data were analysed using a Kruskal–Wallis test with Dunn’s *post-hoc* analysis. Significance was set at p < 0.05.

There was a genotype effect for blood pressure (Fig. 2a; p < 0.001) and shock index (Fig. 2b; p < 0.001) in response to plexiglass-restraint. No differences were detected in blood pressure between male WT, female WT and female *mdx*-het mice (p ≥ 0.111), with all showing a hypertensive response (142–161 mmHg)^21^. Male *mdx*-hemi mice became hypotensive during the plexiglass-restraint (86 mmHg) while *mdx*-homo mice remained normotensive (104 mmHg)^21^. Similarly, hypovolemic shock, or shock index, remained normal and indifferent between male WT, female WT and female *mdx*-het mice (p ≥ 0.208)^21^. Male *mdx*-hemi mice had a two-fold increase in shock index relative to male WT, female WT and female *mdx*-het mice (p < 0.001) and a 1.25-fold increase relative to female *mdx*-homo mice (p < 0.001).

**Figure 3:**
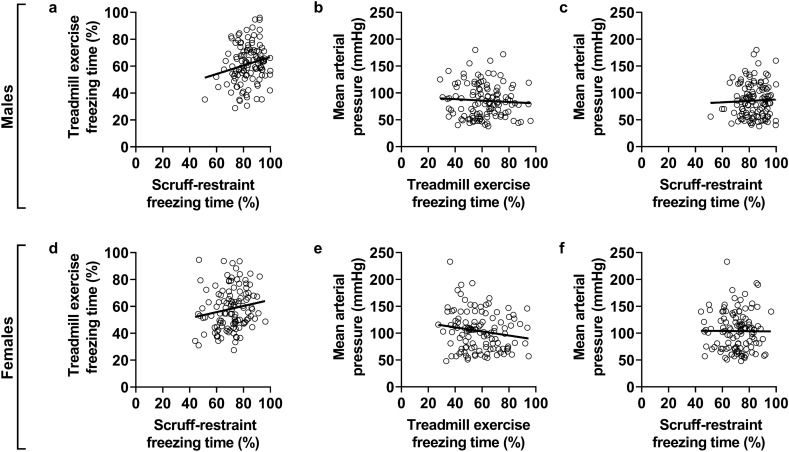
Idiosyncratic response of *mdx* mice to various stressors. (**a**) Relationship between freezing time (%) after treadmill exercise and scruff-restraint in male (*mdx*-hemi) and (**d**) female (*mdx*-homo) *mdx* mice. (**b**) Relationship between mean arterial pressure (MAP) during a plexiglass-restraint stress and freezing time (%) after treadmill exercise in male (*mdx*-hemi) and (**e**) female (*mdx*-homo) *mdx* mice. (**c**) Relationship between MAP during a plexiglass-restraint stress and freezing time (%) after scruff-restraint in male (*mdx*-hemi) and (**f**) female (*mdx*-homo) *mdx* mice. *Mdx*-hemi (n = 119) and *mdx*-homo (n = 117). Data were analysed by linear regression.

Due to the high inter-individual response of *mdx* mice to behavioural stressors (Fig. 1, 2), we wanted to determine if the fear response of individual male *mdx*-hemi and female *mdx*-homo mice was uniform across multiple types of stressors. Using freezing time (%; time spent immobile) after treadmill exercise and scruff-restraint, as well as mean arterial pressure during the plexiglass-restraint, we completed correlative analyses and discovered no relationship in the fear response of male *mdx*-hemi (Fig. 3a–c; R^2^ = 0.001–0.040) and female *mdx*-homo mice (Fig. 3d–f; R^2^ < 0.001–0.027) to various stressors.

**Figure 4:**
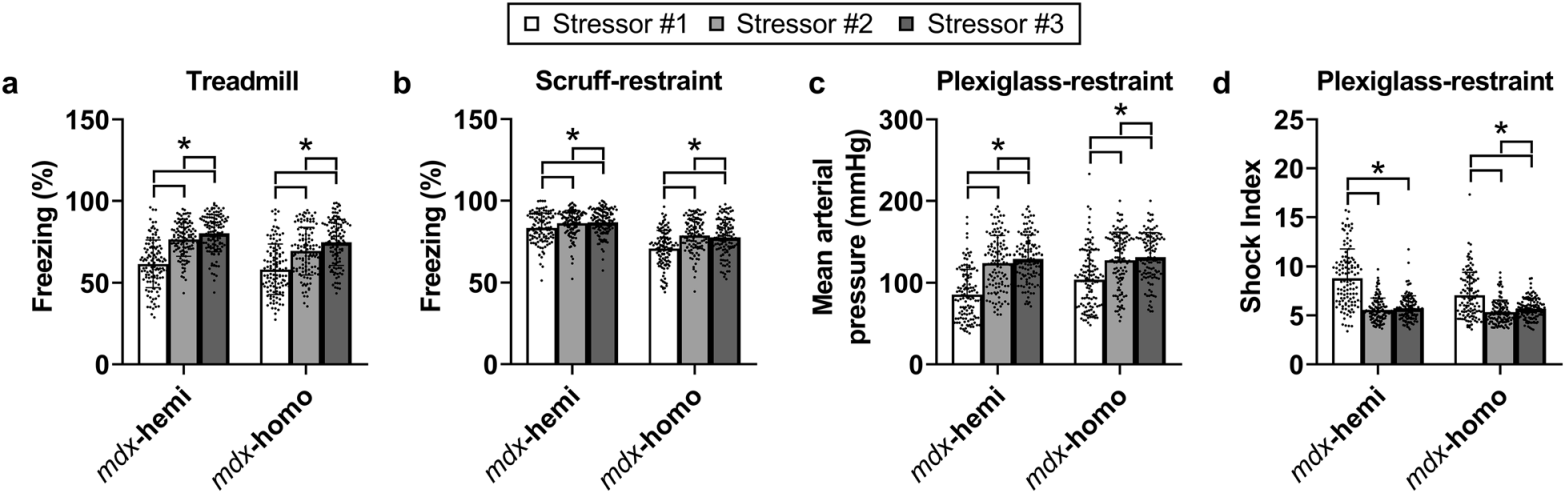
The habituation response of m*dx* mice to stress is stressor specific. (**a**) Freezing percentage (time spent inactive following a stressor) following repeated forced downhill treadmill exercise stress and (**b**) repeated scruff-restraint stress in male (*mdx*-hemi) and female (*mdx*-homo) *mdx* mice. (**c**) Mean arterial blood pressure and (**d**) shock index (heart rate/systolic pressure) during repeated 5 min plexiglass-restraint stress. Stressors were completed 1 week apart. *Mdx*-hemi (n = 119) and *mdx*-homo (n = 117). Data were analysed using a repeated measures two-way ANOVA with Tukey’s *post-hoc* analysis. Data are mean ± SD. *p < 0.05.

When challenged by repeated treadmill exercise and scruff-restraint stressors, male *mdx*-hemi and female *mdx*-homo mice did not adapt (Fig. 4a,b). The level of physical inactivity increased with each successive exposure for both male *mdx*-hemi and female *mdx*-homo mice (p ≤ 0.015), but female *mdx*-homo mice remained more physically active relative to male *mdx*-hemi mice across both stressors (p ≤ 0.014). In contrast, male *mdx*-hemi and female *mdx*-homo mice both became hypertensive following a second plexiglass-restraint exposure (Fig. 4c; 124–128 mmHg; p < 0.001) that did not change after a third (129–132 mmHg; p ≥ 0.300). No differences in mean arterial pressure were detected between male *mdx*-hemi and female *mdx*-homo mice following the repeated exposures (p ≥ 0.783). Similarly, shock index decreased for both male *mdx*-hemi and female *mdx*-homo mice after a second plexiglass-restraint exposure (p < 0.001) that did not change after a third (Fig. 4d; p ≥ 0.538). The shock index for *mdx*-hemi mice remained higher than female *mdx*-homo mice after the second exposure (p < 0.001) but not the third (p = 0.063).

### Study 2

**Figure 5:**
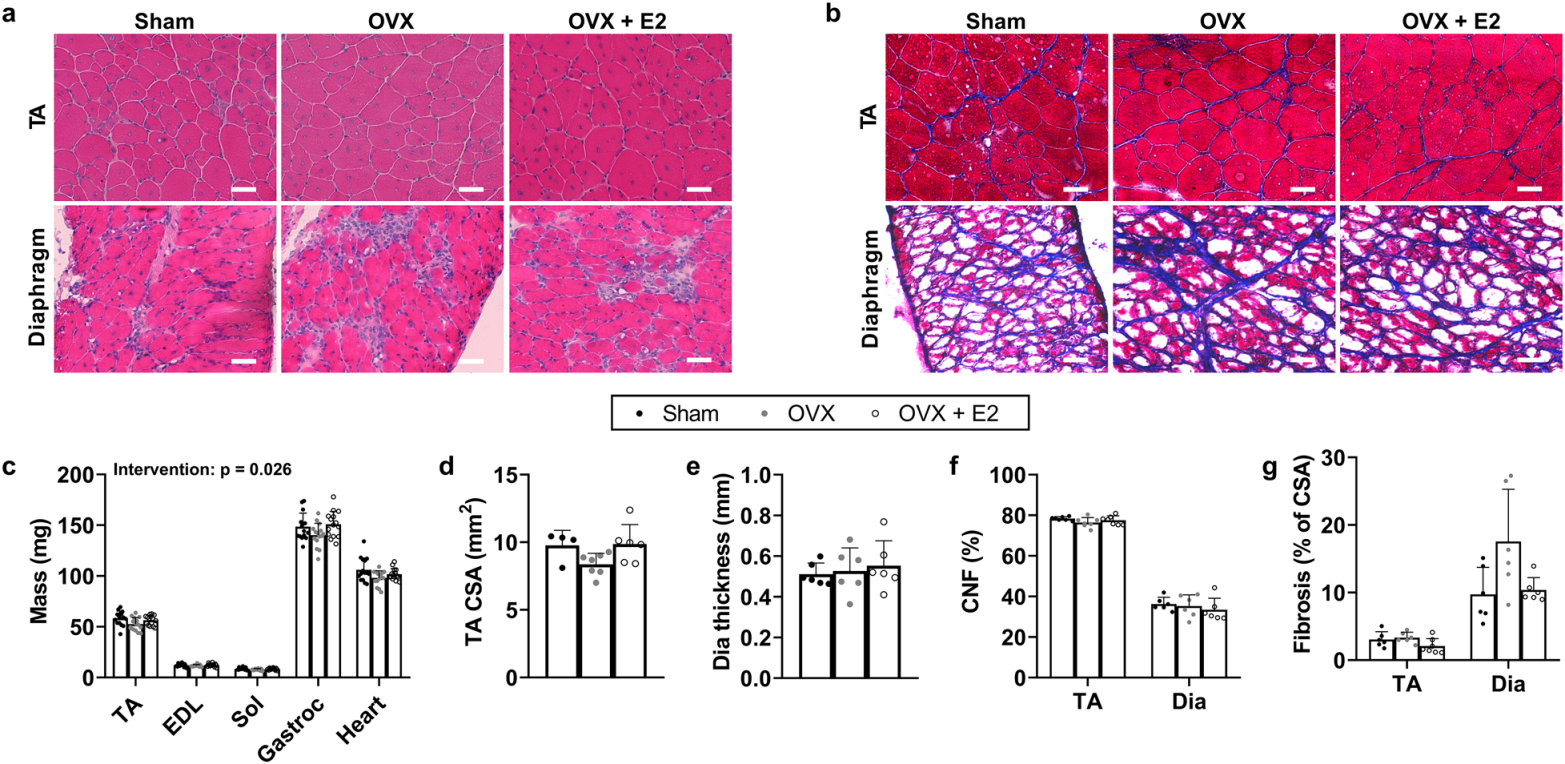
The effect of ovariectomy on skeletal muscle mass, morphology and histology in female *mdx* mice. (**a**) Representative H&E sections and (**b**) trichrome staining for collagen (blue) of the tibialis anterior (TA) and diaphragm muscle in sham-operated (sham; laparotomy without removal of the ovaries), ovariectomized (OVX) and OVX + oestradiol (OVX + E2) female *mdx* mice. (**c**) Striated muscle mass, (**d**) TA cross-sectional area (CSA), (**e**) diaphragm (Dia) thickness, (**f**) central nucleated fibers (CNF) and (**g**) collagen content (fibrosis) in sham, OVX and OVX + E2 female *mdx* mice. N = 5–15/group. Scale bar is 50 µm. Muscle masses were analysed using a repeated measures two-way ANOVA with Tukey’s *post-hoc* analysis, TA CSA, TA CNF, Dia CNF and Dia fibrosis were analysed by Kruskal–Wallis test with Dunn’s *post-hoc* analysis, and TA fibrosis and Dia thickness were analysed by a one-way ANOVA with Tukey’s *post-hoc* analysis. Data are mean ± SD. *p < 0.05.

First, we evaluated the impact of OVX on female *mdx*-homo mice (Fig. 5a,b). There was a significant intervention effect on skeletal muscle mass (Fig. 5c; p = 0.026), with OVX lowering muscle mass relative to sham. The replacement of E2 in OVX mice normalised muscle mass. There was no effect of OVX or OVX + E2 on the cross-sectional area of the TA (Fig. 5d; p = 0.065), thickness of the diaphragm (Fig. 5e; p = 0.779) or number of central nucleated fibers in the TA or diaphragm (Fig. 5f; p = 0.452). OVX, or the replacement of E2 in OVX mice, did not affect collagen content in the TA or diaphragm muscle relative to sham mice (Fig. 5g; p ≥ 0.084).

**Figure 6:**
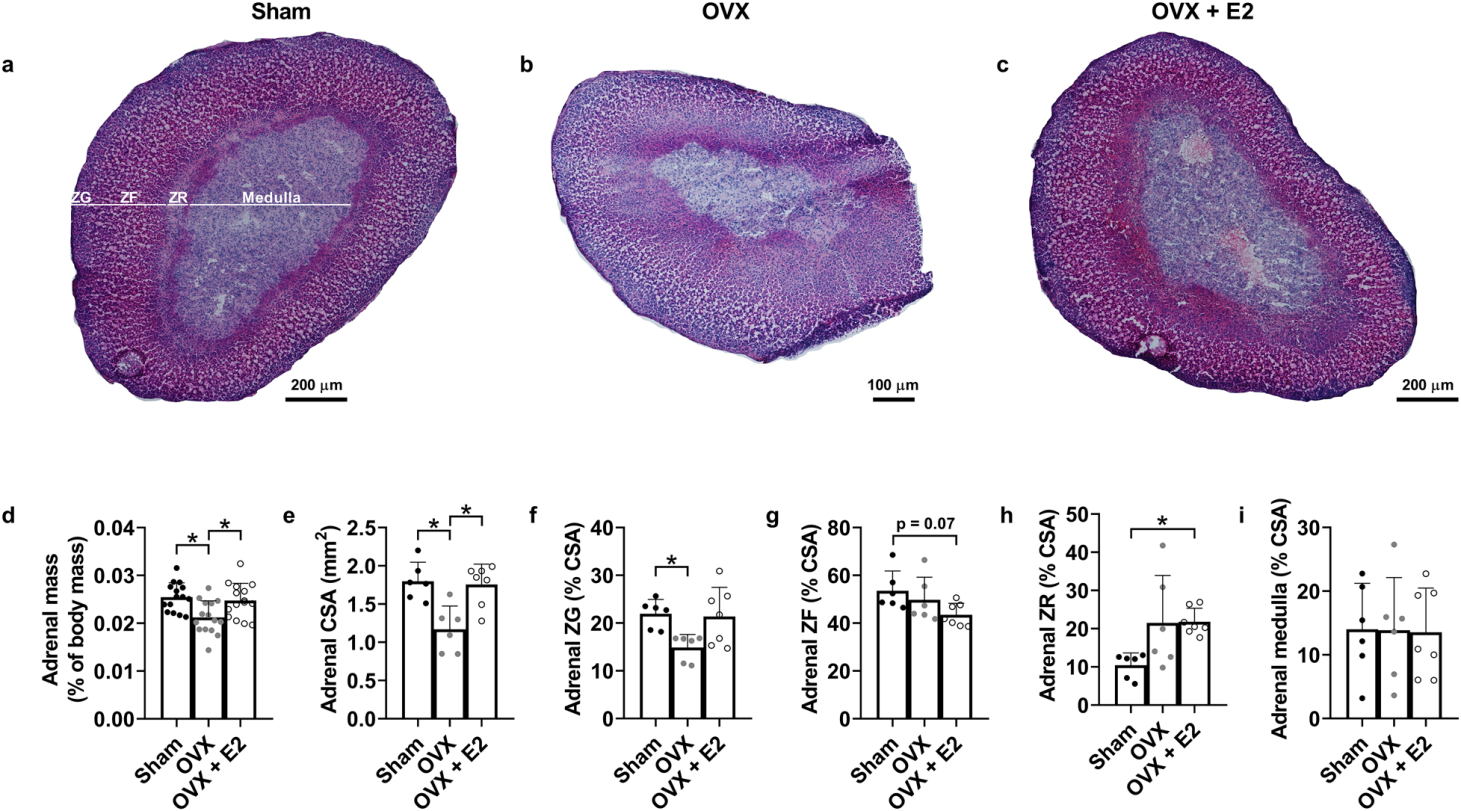
Ovariectomy affects adrenal gland morphology in female *mdx* mice. (**a**) Representative H&E cross-sectional stain of the adrenal gland in sham-operated (sham; laparotomy without removal of the ovaries), (**b**) ovariectomized OVX) and (**c**) OVX + oestradiol (E2) female *mdx* mice. (**d**) Adrenal mass, (**e**) adrenal cross-sectional area (CSA), (**f**) adrenal zona glomerulosa (ZG) area, (**g**) adrenal zona fasciculata (ZF) area, (**h**) adrenal zona reticularis (ZR) area and (**i**) adrenal medulla area in sham, OVX and OVX + E2 female *mdx* mice. N = 5–15/group. Adrenal mass, adrenal CSA, adrenal ZF and adrenal medulla were analysed by one-way ANOVA with Tukey’s *post-hoc* analysis. Adrenal ZG and adrenal ZR were analysed by Kruskal–Wallis test with Dunn’s *post-hoc* analysis. Data are mean ± SD. *p < 0.05.

Adrenal gland activity has been implicated in the stress response of *mdx* mice^18,20,21^ so we assessed the impact of OVX on adrenal gland morphology in female *mdx*-homo mice (Fig. 6a–c). OVX lowered adrenal mass (Fig. 6d; p = 0.003) and adrenal cross-sectional area (Fig. 6e; p = 0.002) relative to sham mice that was normalized with E2 replacement. OVX lowered the zona glomerulosa area that was rescued with E2 (Fig. 6f; p = 0.017) while the zona fasciculata area was not affected by OVX (p = 0.663) and trended toward lower in OVX + E2 mice (Fig. 6g; p = 0.074). OVX did not affect zona reticularis area relative to sham mice (Fig. 6h; p = 0.094) but the OVX + E2 group had greater zona reticularis area relative to sham mice (Fig. 6h; p = 0.008). There were no differences in medulla size between groups (Fig. 6i; p = 0.993).

**Figure 7:**
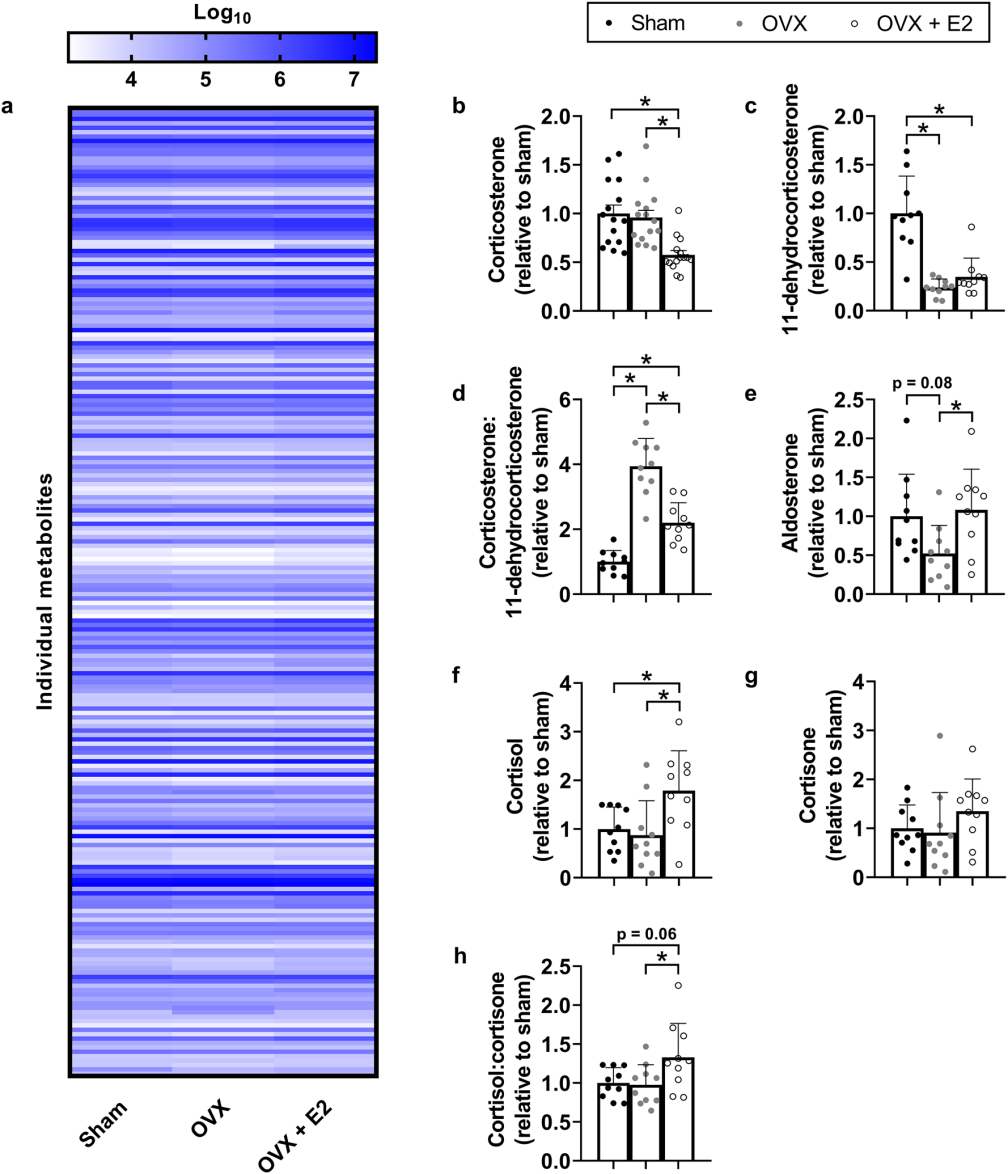
Ovariectomy/oestrogen differentially regulate HPA and RAAS activity in female *mdx* mice in response to stress. (**a**) Metabolomic screen, (**b**) corticosterone, (**c**) 11-dehydrocorticosterone, (**d**) corticosterone:11-dehydrocorticosterone, (**e**) aldosterone, (**f**) cortisol, (**g**) cortisone and (**h**) cortisol:cortisone in serum of sham, OVX and OVX + E2 female *mdx* mice following a 30 s scruff-restraint. N = 10/group. All data were analysed by one-way ANOVA with Tukey’s post-hoc analysis except 11-dehydrocorticosterone and cortisone, which were analysed by Kruskal–Wallis test with Dunn’s *post-hoc* analysis. Data are mean ± SD. *p < 0.05.

To confirm differences in zona glomerulosa and zona fasciculata area between groups, we completed a metabolomic screen on serum collected 15 min following a 30 s scruff-restraint (Fig. 7a; Supplemental Table S1). Serum corticosterone was not affected by OVX but OVX + E2 showed a trend toward a lower concentration relative to sham and OVX (p = 0.07). To confirm the validity of the metabolomic screen, we also selected corticosterone to analyse via ELISA. Serum corticosterone was not affected by OVX but OVX + E2 lowered concentrations relative to sham and OVX (Fig. 7b; p < 0.001). The inactive derivative of corticosterone, 11-dehydrocorticosterone, was lower in OVX and OVX + E2 groups relative to sham (Fig. 7c; p < 0.001) while the corticosterone:11-dehydrocorticosterone was elevated in OVX relative to sham and partially rescued with E2 (Fig. 7d; p < 0.001). This steroidal biosynthesis resulted in a 50% reduction in aldosterone production in OVX mice relative to sham that was normalised in OVX + E2 mice (Fig. 7e; p = 0.031). Interestingly, the non-dominant branch of the HPA axis in rodents, cortisol production, was unaffected by OVX but elevated in OVX + E2 relative to sham (Fig. 7f; p = 0.011). This trend was not evident for the inactive form of cortisol, cortisone (Fig. 7g; p = 0.170), but the cortisol:cortisone was not affected by OVX but elevated by OVX + E2 (Fig. 7h: p = 0.030).

## Discussion

The unconditioned fear or threat response in dystrophin-deficient vertebrates is robust but complex, with several associative mechanisms explaining or rescuing the phenotype^5,17,20–23,27^. Here, our understanding of the fear or threat response in *mdx* mice is expanded by showing a correlative relationship between vascular function, level of dystrophin expression (female *mdx*-het; striated muscle and brain^17,28^) and adrenal function with this behavioural phenotype.

Generation of a hypertensive response to a stressor contributes to an appropriate “fight-or-flight” response in normal physiology. When challenged by a severe stressor, dystrophin-deficiency kills the majority of male *mdx* mice due to sustained hypotension^21^. In this study female *mdx*-homo, relative to male *mdx*-hemi mice, remained normotensive during a stressor. This may explain their greater level of physical activity after stress and the consequence of their HPA and RAAS super activation after a stressor^20^. The hypotensive response of male *mdx*-hemi mice to plexiglass-restraint stress closely resembles blood pressure data from both scruff-restraint and social defeat stress in this mouse model^21^. However, male and female WT mice have a greater hypertensive response to plexiglass-restraint stress relative to scruff-restraint and social defeat. This suggests plexiglass-restraint stress elicits a greater stress response, or variability between the blood pressure tail cuff and radio-telemetry methodologies. Using the non-invasive blood pressure tail cuff method, female *mdx*-het mice, which express 30–60% dystrophin levels in skeletal muscle and brain^17,28^, generate a hypertensive response to stress and present with a WT-like shock index. These data suggest that 30–60% dystrophin expression in skeletal muscle is sufficient to normalise the vascular response to stress in a dystrophin-deficient mouse.

Data from this study indicates that oestrogen is necessary for normal adrenal gland mass, zona glomerulosa area of the adrenal gland and corresponding aldosterone production during a stressor in *mdx* mice. These data are like that described in WT rodents, where ovariectomy lowers adrenal gland mass^29^, decreases adrenal cortex activity^30^ and expands zona glomerulosa cells^30^. Oestradiol also increases aldosterone secretion in female rats^31^. In contrast, oestrogen does not appear necessary for zona fasciculata area in *mdx* mice, which corresponded with a lower corticosterone production during a stressor. This outcome suggests that progesterone, which is also abolished with OVX, is necessary to normalise the area of the zona fasciculata and corticosterone synthesis and that this is necessary to restore the physical activity response to moderate stressors such as scruff-restraint^20^. Interestingly, OVX + E2 mice had elevated levels of cortisol production, which are typically low in rodents but shown to be responsive to stress^32^. This elevation in OVX + E2 mice, relative to sham and OVX groups, may be a compensatory mechanism associated with low levels of corticosterone synthesis. OVX and OVX + E2 also resulted in a larger zona reticularis in female *mdx*-homo mice. This may be a compensatory response to upregulate androgen precursor synthesis and increase sex hormone production. Additionally, oestrogen partially stabilizes HPA activity via restoration of the corticosterone:11-dehydrocorticosterone. This is a response in mice that limits corticosterone production to prevent a heightened and prolonged stress response^33^. Collectively, these data suggest oestrogen, and by process of elimination, progesterone, are required to maintain normal adrenal morphology and function during a stressor that elicits an appropriate response to maintain normotension in *mdx* mice.

The hormonal, adrenal and vascular outcomes in this study support the peripheral hypothesis associated with the robust fear-related behavioural phenotype of *mdx* mice. The peripheral restoration of dystrophin (skeletal muscle-specific) in *mdx* mice prevents lethality during extreme stressors such as social defeat via generation of a hypertensive response^21^. Similarly, restoring dystrophin in skeletal muscle also rescues the unconditioned fear response in *mdx* mice to less severe stressors such as scruff-restraint. On the contrary, the central hypothesis, which centers on dystrophin restoration in the central nervous system^5,22^, partially rescues the response to mild stressors such as brief restraint stress in an expression-dependent manner. The partial rescue of this response is directly associated with brain-specific dystrophin expression and appears dependent on low (10–35% in brain homogenate in Zarrouki et al.^22^) or very low (~ 27% in the postsynaptic density fraction of brain homogenate in Sekiguchi et al.^5^) levels of expression. These studies, combined with the data presented in this study, suggest a relationship between stressor intensity, dystrophin expression levels and the contribution of peripheral and central mechanisms in the unconditioned fear response of *mdx* mice. However, it is unknown if blood pressure is affected by restoration of dystrophin in the central nervous system during a stressor and what impact 10–35% dystrophin expression has on the fear response during more extreme stress protocols. Convoluting these findings are the data presented here on *mdx*-het mice, which express 30–60% dystrophin in the central and peripheral tissues^17,28^ and have a WT-like response to the stressful stimuli in this study. Goyenvalle et al., who restored dystrophin expression in brain and striated muscle in *mdx* mice via intravenous administration of an antisense oligonucleotide made of tricyclo-DNA, also rescued the freezing response to scruff-restraint^34^. Thus, it remains challenging to determine what contribution central and peripheral mechanisms have on the heightened unconditioned fear response of *mdx* mice.

In response to plexiglass-restraint and scruff-restraint, female *mdx*-het mice showed comparable vascular function and physical activity to WT mice, respectively, which corroborates previous findings^17^. However, female *mdx*-het mice were less active than male and female WT mice in response to treadmill exercise. Treadmill exercise, a less severe stressor than a scruff-restraint in *mdx* mice^20^, causes anxiety-like symptoms in rodents^35^. These data suggest that female *mdx*-het have greater levels of anxiety relative to WT mice. Given human female carriers (women who carry a DMD gene mutation in one of their X-chromosomes) show comparable levels of neurocognitive disabilities to boys with DMD, including attention state^36^, these data suggest that anxiety may also be common in this population.

Consistent findings between studies show that *mdx* mice exhibit a hypersensitive fear response to stressors such as treadmill exercise, scruff-restraint, tube-restraint (plexiglass-restraint) and social defeat stress. However, evidence in this study and others^20^ indicate some *mdx* mice demonstrate a WT-like response to various stressors and that this response can be stressor-specific (i.e., a mouse that appears WT-like after scruff-restraint develops hypotension to plexiglass-restraint). Therefore, when designing experiments to assess the response of *mdx* mice to various stressors, either to understand the basic mechanisms of the fear response or to test genetic therapies, large cohorts of *mdx* mice and the inclusion of multiple stressors should be considered.

Male m*dx*-hemi mice appear not to adapt or habituate to daily stress^18,19^, although some adaptation may occur when the stress is applied multiple times within the same day^18^. In accordance with these previous findings, male *mdx*-hemi mice in this study did not adapt to weekly treadmill exercise or scruff-restraint. In fact, male *mdx*-hemi mice were less physically active after each successive stressor indicating their stress sensitivity continually increased. Similarly, female *mdx*-homo mice presented with a comparable response, also becoming increasingly more sensitive to treadmill exercise and scruff-restraint with repeated exposure. These levels of physical inactivity in *mdx* mice are like that observed in WT mice exposed to repeated scruff-restraints, where levels of physical inactivity also increased^18^. In contrast, the physical activity response to stressful stimuli in WT mice, which is dependent on age, also appears to positively adapt when the mouse is exposed to a chronic stress paradigm^37^. Interestingly, when exposed to the plexiglass-restraint stress, male *mdx*-hemi and female *mdx*-homo mice showed an adaptive cardiovascular response. Here, mean arterial blood pressure and shock index became more WT-like with successive exposures. It is plausible that physiological processes such as blood pressure, vs. behavioural manifestations such as physical activity, could have independent responses to stress when dystrophin is absent. A stressor-dependent effect may also influence habituation differences. For example, plexiglass-restraint is the only stressor where the mouse is not physically restrained or forced to exercise but is confined to a tube without human interference. Plexiglass-restraint is also considered less intense than a scruff-restraint when applied for 5 min^18^. In WT mice, mean arterial pressure increases during a stressor and repeated stressors results in a smaller increase in mean arterial pressure with successive stimuli, which is accompanied by a faster rate of recovery after the cessation of the stimuli^38^. These data suggest that while behaviour of dystrophin-deficient male and female mice does not adapt to repeated stress, physiological adaptations such as blood pressure could mitigate the potential for life-threatening episodes that mimic adaptations observed in WT mice.

The ovariectomy performed in this study was for 4–5 weeks, a period sufficient to lower striated muscle masses that was rescued with E2. The loss of muscle mass and rescue with E2 are like data generated in WT rodents and humans^39,40^. These data suggest that in a dystrophin-deficient environment, loss of the female sex hormones, like that observed during menopause, negatively impacts skeletal muscle size. In human female carriers, a spectrum of skeletal muscle weakness is observed^41,42^, that may progress with age^41^ and parallel a natural decrease in oestrogen availability. However, to the best of our knowledge, no data has indicated that menopause impacts muscle mass and/or strength in human female carriers, perhaps due to the large variability in skeletal muscle dystrophinopathies in this population^43,44^. Other indices of dystrophinopathy, such as central nucleated fibres, muscle thickness and muscle fibrosis, were not affected by ovariectomy. These data are in accordance with similar studies showing no difference in central nucleated fibres^45^ or collagen content^46^ in OVX female *mdx*-homo mice. This suggests that loss of the female sex hormones, for short periods of time (3–5 weeks), does not dramatically impact skeletal muscle pathology phenotypes of dystrophinopathy.

## Conclusions

The unconditioned fear response in *mdx* mice is associated with an inability to generate a hypertensive response. This response appears normalised in female *mdx*-het mice (30–60% dystrophin expression in brain and striated muscle^17,28^) and is associated with an oestrogen-dependent relationship with adrenal gland morphology and function.

## Supplementary Information

Supplementary Information.

## Data Availability

Data will be made available upon reasonable request of the corresponding author.
